# The effect of an escape room game on college nursing students’ learning attitude and game flow experiences in teaching safe medication care for the elderly: an intervention educational study

**DOI:** 10.1186/s12909-023-04961-3

**Published:** 2023-12-12

**Authors:** Dong Chen, Fang Liu, Chongkuan Zhu, Chunling Tai, Yuhuan Zhang, Xiaoyu Wang

**Affiliations:** 1 Department of Nursing, Heilongjiang Nursing College, 209 Xuefu Road, Harbin, Heilongjiang Province 150086 China; 2 Intensive Care Unit, Qingdao Hiser Hospital Affiliated of Qingdao University (Qingdao Traditional Chinese Medicine Hospital), 4 Renmin Road, Qingdao, Shandong Province 266000 China; 3 Intensive Care Unit, Qingdao Hiser Hospital Affiliated of Qingdao University (Qingdao Traditional Chinese Medicine Hospital), 4 Renmin Road, Qingdao, Shandong Province 266000 China; 4 Department of Ophthalmology, The Second Affiliated Hospital of Harbin Medical University, 246 Xuefu Road, Harbin, Heilongjiang Province 150086 China; 5 Student Section, The Second Affiliated Hospital of Harbin Medical University, 246 Xuefu Road, Harbin, Heilongjiang Province 150086 China; 6 Department of Emergency, Qingdao Hiser Hospital Affiliated of Qingdao University ( Qingdao Traditional Chinese Medicine Hospital), 4 Renmin Road, Qingdao, Shandong Province 266000 China

**Keywords:** Education, Nursing, Teaching methods, Gerontological nursing, Escape room, Learning attitude, Game Flow Experience

## Abstract

**Background:**

In the context of an aging population, the Gerontological Nursing course is becoming more and more important. Escape room games have been shown to have a positive effect on nursing education, but they have not been applied in the Gerontological Nursing course. The purpose of this study was to investigate the effects of adding an escape room game-based learning activity at the end of classroom teaching in a Gerontological Nursing course on nursing students’ learning attitude and game flow experience.

**Methods:**

In April 2023, a total of 84 nursing students from two classes at a medical school in Northeast China were selected for the study, and the classes were divided into a test group (n = 41) and a control group (n = 43). Both groups received regular classroom teaching on “Safe Medication Care for the Elderly”, and the test group participated in an escape room game at the end of the classroom teaching. General information about the nursing students in both groups was collected prior to participation; learning attitude were measured before and after participation; and game flow experience was measured before and after participation in the test group. Data were analyzed using independent samples t-tests and paired samples t-tests.

**Results:**

There was no statistically significant difference in the general information and learning attitude of nursing students between the two groups before participation. After participation, the total learning attitude score of nursing students in the test group was (73.17 ± 1.67) and that of the control group was (61.63 ± 2.66), and the difference was statistically significant (*p* < 0.001, Cohen’s d = 5.196). The game flow experience of nursing students in the test group before and after participation was (63.27 ± 2.48) and (81.29 ± 2.49), respectively, and the difference between before and after was statistically significant (*p* < 0.001, Cohen’s d = 5.253).

**Conclusions:**

During the teaching process of the Gerontological Nursing course, an escape room game added at the end of classroom teaching can improve nursing students’ learning attitude and also help them to have a good game flow experience. These findings suggest that teaching activities based on the escape room game have considerable practical application value.

**Supplementary Information:**

The online version contains supplementary material available at 10.1186/s12909-023-04961-3.

## Introduction

Population aging is a serious phenomenon worldwide, and China is one of the countries with the fastest rate of population aging. The degree of aging will continue to deepen in the coming time [1]. There will also be a serious shortage of geriatric nursing personnel, and vigorously cultivating geriatric nursing personnel has become one of the important training tasks of the current nursing profession [2]. Geriatric nursing-related courses, represented by the Gerontological Nursing course, are gradually becoming the core curriculum of the current nursing program [3, 4]. Compared to other courses, Gerontological Nursing is less popular with nursing students [5]. On the one hand, junior college nursing students currently studying are generally millennials, with distinct personality traits that make them dislike traditional teaching methods and prefer immersive learning experiences [6]. On the other hand, nursing students are younger, and there is a huge age gap and generational differences between them and older adults [7],which makes it difficult for nursing students to relate to the real physical and mental conditions of older adults and to empathize with them, and thus they are less interested in learning in Gerontological Nursing course, have a lower sense of classroom engagement, and even experience rejecting academic sentiments [8].

### Escape room game teaching

An escape room (ER) game is a commercial game that usually consists of a team of several persons discovering and puzzling multiple game elements in an enclosed space and a set amount of time to successfully complete a defined task [9]. In recent years, ER games have gradually been applied in the field of medical teaching and have shown positive effects on teaching in clinical medicine [10], pharmacy [11], dentistry [12], imaging medicine [13], and nursing [14]. In the field of nursing teaching, ER games have been shown to increase nursing students’ learning satisfaction [15], promote communication [16], enhance teamwork [17], and critical thinking [18]. Although the existing research literature has shown that ER game have a positive impact on nursing education, few studies have examined the effectiveness of games for teaching courses related to Gerontological Nursing.

The Gerontological Nursing course is one of the required courses for college nursing students. It not only contains theoretical knowledge of various common and unique diseases of the elderly but also includes a variety of basic nursing skills commonly used by the elderly. However, nursing students’ lack of interest in learning the content of the course and insufficient knowledge recognition currently call for exploring and experimenting with delivery methods that enhance nursing students’ sense of learning experience. As a solution, this study designed an ER game based on the theme of “Safe Medication Care for the Elderly” and conducted it at the end of traditional classroom teaching in order to help nursing students gain an immersive learning experience.

### Learning attitude and game flow experience

Learning attitude is a relatively stable psychological tendency shown by nursing students towards nursing professional course learning and its learning situation, which is a comprehensive evaluation of nursing students’ knowledge, experience, and behavioral tendency towards the current learning activities [19]. It is obvious that the learning attitude can directly judge the degree of recognition of nursing students towards the content of the course of study and its effect. Despite the importance of learning attitude, research [20] has found that nursing students’ learning attitude is not encouraging. Game flow experience refers to an emotional experience that occurs when an individual shows a strong interest in an activity or thing and is completely devoted to that activity or thing, and when people are in a flow state of mind, they usually show too much attention and obliviousness [21]. In this case, a good game flow experience is one of the manifestations of highly immersive learning for nursing students.

## Methods

### Aims

This study sought to address two gaps in research on ER game in nursing education: (1) the lack of research on teaching topics related to Gerontological Nursing; and (2) the lack of research related to learning attitude and the game flow experience. We examined the role of ER game in improving nursing students’ learning attitude in a Gerontological Nursing course, as well as a performance aspect of immersive learning, the game flow experience. A prospective intervention study was conducted to determine the effects of an interventional educational activity based on an ER game on nursing students’ learning attitude and the game flow experience after they had received nursing classroom teaching on safe medication use in older adults. The purpose of the study was to test two hypotheses:

1. The ER game would have a more positive impact on nursing students’ learning attitude compared with a traditional classroom-based teaching group.
2. In the post-intervention test, the pilot group of nursing students had a better sense of the game flow experience than the pre-intervention one.

### Participants

This is a prospective intervention educational study. In April 2023, we conducted the study among nursing students at a medical school in northeastern China. Prior to the start of the study, we invited counselors and all class presidents to a research workshop and recruited 83 current nursing students based on their wishes. They were all nursing students in two classes, currently in their second year of college, had completed part of the Medical Nursing and Surgical Nursing courses, and were taking the Geriatric Nursing course. The test group class (n = 41) and the control group class (n = 43) were identified by a simple lottery method. All nursing students were informed about the purpose, methodology, potential benefits, and risks of the study and signed a consent form.

### Study design

During the study period, both groups of nursing students received classroom teaching in the “Geriatric Nursing” course, which was in accordance with the lecture program that had been established prior to studying the course. In the Gerontological Nursing course, the chapter “Safe Medication Care for the Elderly” contains more important knowledge points such as the characteristics and principles of medication use in the elderly, guidance on medication use in the elderly, characteristics of common adverse drug reactions in the elderly, and nursing measures for the safe use of medications. After both test and control group nursing students received classroom teaching by the same teacher, the test group nursing students participated in an additional ER game. After completing all questionnaire measurements for both groups of nursing students, the control group of nursing students also participated in the same additional ER game.

### ER game design and procedures

#### Preliminary

(1)Formation of a project team: the director of the teaching and research department and three lecturers formed a project team, and they participated in an ER game of a commercial nature together. The teachers in the group experienced the game scenarios, game elements, and link settings of the escape game and gained the experience of participating in the escape game. Afterwards, they worked together to design an escape game with the theme of.

Safe Medication Care for the Elderly. (2) Adequate review of literature: by combing the literature related to the ER game in the field of teaching and learning at home and abroad [22–26], to further design the steps and links of the ER game. (3) Venue selection: The geriatric nursing training room was selected as the venue for the ER game. The geriatric nursing training room is an experience venue for senior citizens and contains scenes of the daily lives of the elderly, which is suitable for the ER game. (4) Pre-experimentation: Teachers outside the project team were asked to experience the escape game in advance, to find out the risk points and inappropriate connections in each part of the game in advance, and to further improve and perfect them. The pre-experiment found that it took about 25 min for the teachers to complete a complete escape game and about 10 min to recover the scene. (5) Early release of the story background: Referring to the previous literature [15], the teacher informed the nursing students about the story background of the escape game in advance, and the story background of this study was “Dear White Angels, I have a long-time friend, Bennett, and this morning, when we were chatting on the cell phone as usual, his voice suddenly came to a screeching halt. Bennett usually lives alone, and I am concerned that he is unconscious in his own home. There is a small box in the center of his kitchen table that contains his oral medication, but it is locked up with a combination lock, so please try to find the correct combination to save Bennett through your own efforts. As far as I can tell, there are still 40 minutes until the medication wears off, so please act quickly.” The final design of the ER game based on the theme “Safe Medication Care for the Elderly” is available as Additional File 1 and Additional File 2.

#### Implementation process

Nursing students in the test group voluntarily formed a team, 6 ~ 8 nurses as a group. The final test group of nurses formed a total of 6 groups, in accordance with the drawing of lots to decide to enter the secret room serial number. All the nursing students in the test group were concentrated in one classroom and handed in their cell phones. The game site was set up as a one-way passage, and all the nursing students could not return to the original way after participating in the game. In the center of the chamber, a teacher acted as a referee to control the time accurately and give clues to the nursing students when necessary. It took 20 min to restore the chamber after each group.

### Instruments

Paper questionnaires were required to be submitted by both groups of nursing students prior to the start of the ER game in order to collect general information. The completion of the ER game and the time taken by the nursing students in the test group were directly observed and provided by the teachers on site. Nursing students in both groups collected learning attitude questionnaires before and after the start of the ER game. Nursing students in the test group collected the game flow experience questionnaire before and after participating in the ER game.

(1) The general information questionnaire in this study included gender, age, home location, and number of siblings for both groups of junior college nursing students.

(2) Completion of the ER game by students in the test group, i.e., whether they succeeded in solving the puzzles, the time spent, and the route completed.

(3) Learning Attitude Scale (LAS) The Learning Attitude Scale was developed by Yunhan Zhang [27] et al. This scale includes 23 items in four subscales: learning interest, learning experience, learning habit, and professional recognition. Response options range from “absolutely disagree” (1 point) to “absolutely agree” (4 points). Total scale scores range from 23 to 92. Higher scores indicate a better learning attitude among junior college nursing students. The Cronbach’s α for the scale was 0.927.

(4) Game Flow Experience Questionnaire (GFEQ) The Game Flow Experience Questionnaire was developed by Lin Yan [28]. This questionnaire includes 19 items in five subscales: a sense of control, telepresence, a distorted sense of time,enjoyable feelings, and being unconscious of irrelevant surroundings. Response options range from “absolutely disagree” (1 point) to “absolutely agree” (5 points). There are two entries scored in reverse. The total scale of the questionnaire ranges from 19 to 95. Higher scores indicate a better game flow experience for junior college nursing students’ feelings. The Cronbach’s α for the questionnaire was 0.859.

### Statistical methods

Descriptive statistics were used to describe the general information of the nursing students. Independent samples (t-test and chi-square test) were used to compare whether there was any difference in the general information of the nursing students in the two groups. An independent sample t-test was used to compare the differences in learning attitude scores between the two groups of nursing students. A paired sample t-test was used to compare the differences in the scores of the nursing students’ game flow experience before and after participating in the ER game in the test group. *p* < 0.05 was considered statistically significant. All statistical analyses were performed using IBM SPSS ver. 25.0 (IBM Corp., Armonk, NY, USA).

## Results

### Demographic variables of the two groups

Table 1 shows the demographic data of the junior college nursing students who participated in this study. There was no significant difference in age, gender, family location, or number of siblings between the test group and the control group in this study.

**Table 1:** Demographic characteristics of the participates in the sample

Item	Test group	Control group	Statistics	***P***-value
Gender				
Male	9	12	*χ2 =* 0.397	0.529
Female	32	31		
Age	19.88 ± 0.60	19.30 ± 0.70	*t=*-0.365	0.716
Family location				
City	15	17	*χ2 =* 0.077	0.781
Rural	26	26		
Number of siblings				
Zero	24	28	*χ2 =* 0.385	0.535
At least one	17	15		

### Overall escape room completion for nursing students in the test group

Among the 6 groups of nursing students in the test group, 1 group was unsuccessful in solving the puzzle, and 5 groups succeeded in solving the puzzle. The 1 group that was unsuccessful in solving the puzzle did not read the contents of the diary in Scene 2 and answered the questions on the closet directly, and after obtaining the floral mirror, they did not know what to do next, and the final time was over. The 5 groups of nurses who successfully solved the puzzles spent 31 min, 37 min, 33 min, 29 min, and 36 min, respectively, with an average time of 33.2 min; among them, 3 groups realized Route 1 and 2 groups realized Route 2 (none of them missed Task 1).

### Comparison of scores on learning attitude scale between two groups of junior college nursing students

Table 2 (It is placed at the end of the document text file) shows the learning attitude scores of nursing students in the two groups. Before participating in the ER game, there was no difference in the scores and dimensions of the learning attitude scale between the two groups of nursing students, and after participation, the total scores of the learning attitude scale, the learning interest dimension, the learning experience dimension, and the learning habit dimension of the test group were higher than those of the control group (*p* < 0.01), and no difference was seen in the professional recognition dimension.

**Table 2:** Comparison of total scores and dimensions of the learning attitude scale between two groups of junior college nursing students before and after the implementation of the ER game(Mean ± SD)

Group	Learning interest		Learning experience		Learning habit	
	Pre‑test score	Post‑test score	Pre‑test score	Post‑test score	Pre‑test score	Post‑test score
Test group	17.05 ± 1.38	20.29 ± 0.93	14.61 ± 1.02	20.00 ± 0.95	19.29 ± 1.01	22.63 ± 1.16
Control group	17.44 ± 1.61	17.16 ± 1.25	14.84 ± 0.92	15.30 ± 1.04	19.49 ± 0.96	19.26 ± 1.24
*t*	−1.417	12.962	−1.071	21.644	−0.912	12.918
*P*-value	0.160	＜0.001	0.287	＜0.001	0.364	＜0.001
Cohen’s d	0.260	2.841	0.237	4.719	0.203	2.807
	Professional recognition		Learning attitude			
Group	Pre‑test score	Post‑test score	Pre‑test score	Post‑test score		
Test group	9.98 ± 1.21	10.24 ± 1.18	60.93 ± 2.33	73.17 ± 1.67		
Control group	9.74 ± 0.90	9.91 ± 0.87	61.51 ± 2.32	61.63 ± 2.66		
*t*	0.995	1.497	−1.152	23.653		
*P*-value	0.323	0.138	0.253	＜0.001		
Cohen’s d	0.225	0.318	0.249	5.196		

### Comparison of game flow experience questionnaire scores before and after the test group of junior college nursing students participated in the ER game

Table 3 (It is placed at the end of the document text file) shows the game flow experience scores of nursing students in the test group. The total score of the game flow experience questionnaire and all dimensions of the nursing students in the test group were higher after participating in the ER game than before participation, and the difference was statistically significant (*p* < 0.01).

**Table 3:** Comparison of total scores and dimensions of the Game Flow Experience Questionnaire for students in the test group before and after participation(Mean ± SD)

Item	Pre‑test score	Post‑test score	t	***P***-value	Cohen’s d
A sense of control	15.88 ± 1.03	20.85 ± 1.23	-23.59	＜0.001	3.684
Telepresence	14.78 ± 0.99	17.46 ± 0.87	-14.41	＜0.001	2.250
A distorted sense of time	9.51 ± 1.03	12.88 ± 0.78	-17.10	＜0.001	2.671
Enjoyable feelings	9.41 ± 1.02	12.88 ± 0.93	-17.24	＜0.001	2.692
Being unconscious of irrelevant surroundings	13.68 ± 1.08	17.22 ± 0.91	-15.08	＜0.001	2.355
Game flow experience	63.27 ± 2.48	81.29 ± 2.49	-33.63	＜0.001	5.253

## Discussion

### Participation in ER games enhances learning attitude of junior college nursing students

This study found that the total score of learning attitude of nursing students in the test group was significantly higher than that of nursing students in the control group after participating in the ER game (*p* < 0.01), suggesting that teaching activities based on the er game can improve the learning attitude of nursing students. In terms of specific dimensions, there were statistical differences in the dimensions of learning interest, learning experience, and learning habits. On the one hand, the ER game is initially a commercial game, customer loyalty is a prominent feature, and after combining it with the knowledge of “Safe Medication Care for the Elderly”, it can provide nursing students with an interesting and rich learning experience [18], which can increase their interest in learning and prompt them to have a pleasurable experience or a sense of achievement in the learning process. On the other hand, after the combination of the ER game and professional knowledge, fully mastering the professional knowledge becomes the key to the nursing students’ successful puzzle solving, which is conducive to the nursing students’ timely review of the key and difficult knowledge and helps the nursing students establish the correct study habits. This study found that there was no statistical difference between the two groups of nursing students in the dimension of professional recognition, which may be attributed to the fact that the awareness of treating the nursing profession needs to be carried out throughout the whole process of talent cultivation [29], which cannot be changed by participating in an ER game for a short period of time, and therefore there was no difference in the dimension of professional recognition.

### Participation in ER games significantly improves game flow experience of junior college nursing students

In this study, we found that the scores on the game flow experience questionnaire and the scores on all dimensions of the test group of nursing students were higher than those before participation in the ER game (*p* < 0.01), suggesting that participation in the game can enhance the game flow experience of nursing students. According to the theory of flow [30], individuals who engage in their favorite things may experience a unique experience that often leads to sleeplessness, total commitment, and enjoyment regardless of the rewards. The higher than pre-participation scores on the sense of control dimension suggest that the nursing students were more confident in the ER game process and believed that their group would win first place. The higher scores on the telepresence dimension than the pre-participation dimension suggest that the students were able to empathize with the game content and were able to participate in the game situation. When the nursing students in the test group were in a state of “forgetfulness” during the game, they often felt that time was passing quickly and therefore scored higher on the distorted sense of time dimension than they did before participation. The higher scores on the dimension of enjoyable feelings than before indicated that the ER game could make the nursing students feel excited, physically and mentally happy, and have the idea of playing the game all the time. Nursing students subjectively do not want to be disturbed when they are highly focused on the game process, and at the same time, their awareness of irrelevant things around them will be weakened, and the dimension of being unconscious of irrelevant surroundings scored higher than that of the pre-participation period.

### Reflections and suggestions on teaching experience

In this study, an ER game was added at the end of the classroom teaching in the course of Gerontological Nursing. Although a lot of preparations and teaching design were carried out in the early stages, some problems were still revealed during the implementation, such as: some groups of nursing students had disagreements or even quarreled during the game; the progress of the game was slow and stagnant due to the lack of effective leaders in some groups; as only one teacher was set up in the escape room scenario, she needed to maintain order and regulate the progress as well as observe the performance of the nursing students, which led to the teacher’s fatigue; after the activities of the first few groups, some items in the chamber were damaged, resulting in a prolonged restoration of the chamber environment and a decrease in the reuse of teaching tools. In the future, relevant studies can further strengthen the research design or pre-tests by incorporating the above issues, such as setting two or more teachers in the chamber, reserving a longer recovery time for the chamber, and backing up items for the chamber elements.

In addition, recommendations for future research include the following: (1) Teachers need to have the ability to design game sessions for ER games. This study confirms that an additional ER game session at the end of classroom teaching can improve students’ learning attitude. However, professional teachers usually do not know how to design game sessions. This study is based on the foundation of literature research, and the teachers of the group actually participated in ER games of commercial nature to gain some experience. It is recommended that they also refer to commercial ER games or invite professional game designers to participate in the future before designing. (2) Teachers need to think about how to integrate pedagogical goals or specialized knowledge with ER games [31]. ER games are interesting, but how to integrate them with the teaching objectives and how much proportion of professional knowledge is set in the link is still a problem that needs to be thought about. If the ER games and the teaching objectives of the rigid combination or the game process are filled with too much obscure professional knowledge, they not only can’t achieve the expected results of teaching and learning but are also prone to cause students to be disliked.

## Conclusions

This study found that participation in an ER game at the end of classroom teaching during the Gerontological Nursing course improved nursing students’ learning attitude and increased their gaming flow experience. This finding confirms that the pedagogical effect of the ER game remains significant in the context of saving teaching resources and time costs. There are no previous studies exploring the effects of escape room games on learning attitude and game flow experience, and the results of this study suggest ER games should be further promoted in the field of teaching the Gerontological Nursing course, and this teaching method may have a positive impact on the content and learning process of nursing students in courses related to Gerontological Nursing. The implementation experience of this study can be used in the future to further explore the best practice methods and other application effectiveness of the ER game in the Gerontological Nursing course.

### Electronic supplementary material

Below is the link to the electronic supplementary material.

Supplementary Material 1

Supplementary Material 2

## Data Availability

The datasets during and/or analyzed during the current study are available from the corresponding author on reasonable request.

## Abbreviations

ER: escape room
