# Supplementary material for: The effect of an escape room game on college nursing students’ learning attitude and game flow experiences in teaching safe medication care for the elderly: an intervention educational study
Source: BMC Med Educ. 2023 Dec 12;23:945. doi: 10.1186/s12909-023-04961-3 (PMC10717663; doi:10.1186/s12909-023-04961-3)
Supplement: Supplementary file 1 — Supplementary Material 1 [file 12909_2023_4961_MOESM1_ESM.doc]

**Additional File 1
Table 1 Design of ER game sessions on the theme of Safe Medication Care for the Elderly**

| **Topic** Safe Medication Care for the Elderly | **Background** Grandpa Bennett woke up in the morning and suddenly collapsed in his bedroom while chatting with an old friend, and the cause of the coma is unknown. | **Final goal** Get the right password to open the small box on the kitchen table and get the oral medicine to save Grandpa Bennett's life. |
| --- | --- | --- |
| **Scene 1 Bedroom Scene**  **Environmental Setting** Complete bed unit, simulator (dressed), cell phone next to pillow, nightstand (drawers locked and titled), books, poster trash can, TV (projector), and other daily necessities.  **Critical Elements：**cell phone next to pillow, books, poster, drawers locked, TV (projector) | | |
| **Clue 1** Grandpa Bennett's cell phone chat with a friend suggests Grandpa Bennett just purchased a favorite poster a week ago. | **Task 1** Complete the multiple-choice questions in the lower right corner of the poster to get the letters of the alphabet, which combine to form the English word BOOKS. | **Point 1** Dosage forms of commonly used oral medications for the elderly and principles of drug selection |
| **Clue 2** Look at one of the many books on Grandpa Chang's nightstand. The title page of the first book posed the question, and each of the remaining books was interspersed with a puzzle piece, with the correct or incorrect answer written on the back of each piece. | **Task 2** Complete the questions on the title page of the first book, and the puzzle corresponding to all the answers can be put together as a drawer image. | **Point 2** Reasons affecting accurate medication taking in the elderly |
| **Clue 3**.Only after the nursing student identifies the drawer clues is the field instructor given the opportunity to hang more than 20 similar keys, each numbered, on plastic string. Ten multiple-choice questions are taped to the drawer, with the answer to each question having a pointer to the serial number of the next question. | **Task 3** Participating nursing students are not required to answer all 10 questions correctly, but are required to answer questions with pointers correctly in order to receive the correct key number to open the drawer at the end. | **Point 3** Methods of medication care for the elderly with various special diseases |
| **Clue 4** Open the drawer to get the remote control for the projector, turn on the projector, and the drug name crossword appears. | **Task 4** Similar to the idiom game, the nursing students only have to fill in all the drug names accurately for the projector to show the answer to the riddle: DIARY. | **Point 4** Names of commonly used drugs for the elderly and principles of application |
| **Scene 2 Study room scene**  **Environmental Setting** Complete bookcases, desks and chairs, assorted books, journals, pen holders, pokers, closets (combination locks), trash cans, and other daily necessities.  **Critical Elements** Journals, trash cans, poker, closet, reading glasses | | |
| **Clue 5** There are journals on the desk and in the trash can, and the bedside table diary records to the point that Grandpa Lee was bleary-eyed and had to wear a pair of fancy glasses kept in a closet with a combination lock when he played poker and read the diary. | **Task 5** A copy of the test is posted on the closet cabinet; a copy of the answer is glued to the back of each of the nightstand playing cards, and only by choosing the correct answer can you obtain the corresponding playing card number, i.e., the combination cabinet code. | **Point 5** Common adverse drug reactions and characteristics in the elderly |
| **Clue 6** Open the combination cabinet to get the reading glasses and wear them to observe the journal in the trash can in order to discover the Sudoku game written in highlighter. | **Task 6** Combine the numbers in the Sudoku with multiple questions, and only choose the correct answer to calculate the Sudoku and ultimately get the final combination box password. | **Point 6** Adverse drug reactions and first-aid treatment of common human medications in the elderly |
| **Scene 3 Kitchen Scene**  **Environmental Setting** Daily kitchen setup, center placed dining table (combination locking locket)  **Critical Elements** Locket with combination lock | | |
| **Final route** Get the code and successfully open the combination box to get the medicine that saved Grandpa Bennett's life. | | |
| **Possible route**  **Route 1：**Task 1→Task 2→Task 3→Task 4→Task 5→Task 6→Success  **Route 2：**Task 2→Task 3→Task 4→Task 5→Task 6→Success（Completing task 1 midway or missing task 1） | | |
