# Supplementary material for: The effect of an escape room game on college nursing students’ learning attitude and game flow experiences in teaching safe medication care for the elderly: an intervention educational study
Source: BMC Med Educ. 2023 Dec 12;23:945. doi: 10.1186/s12909-023-04961-3 (PMC10717663; doi:10.1186/s12909-023-04961-3)
Supplement: Supplementary file 2 — Supplementary Material 2 [file 12909_2023_4961_MOESM2_ESM.docx]

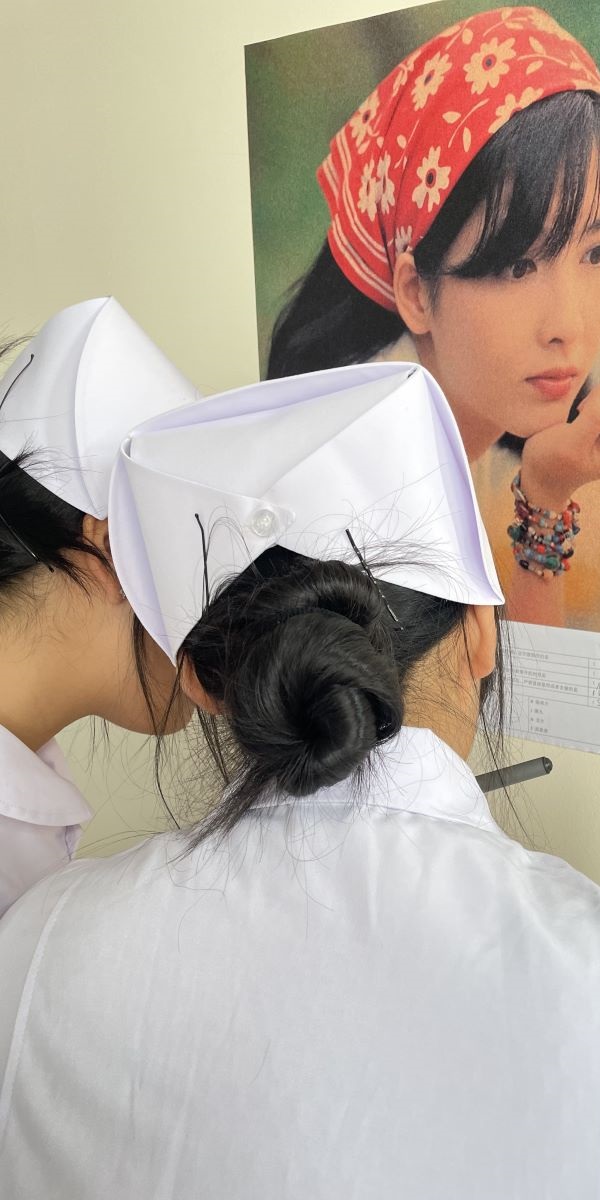


Fig. 1 The questions on the poster


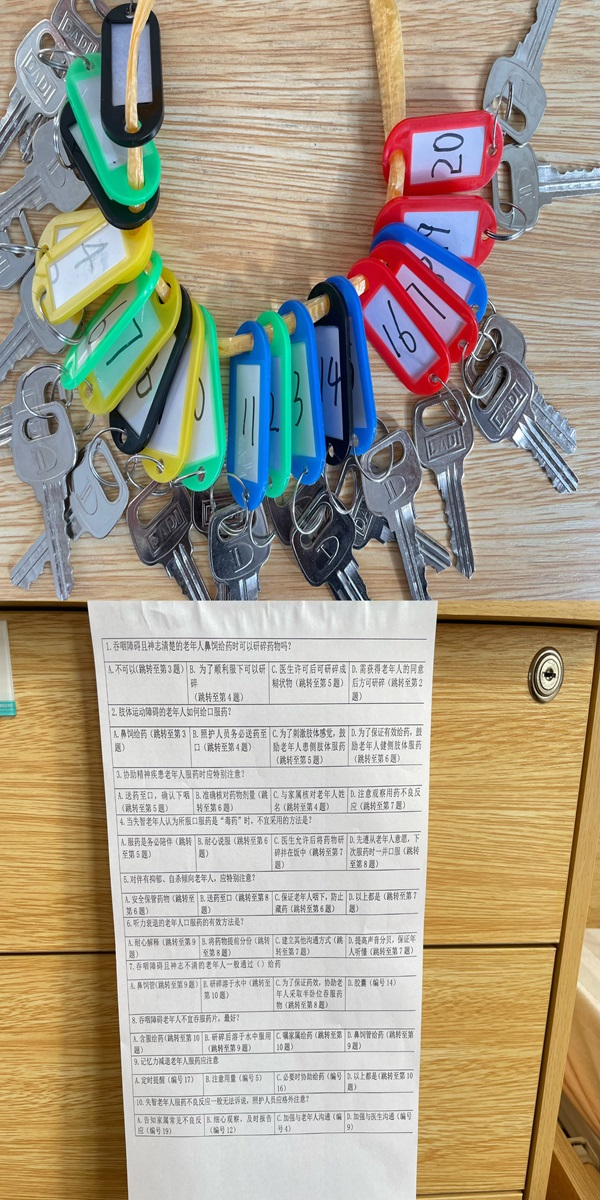


Fig. 2 Multiple choice questions with key numbers and directions


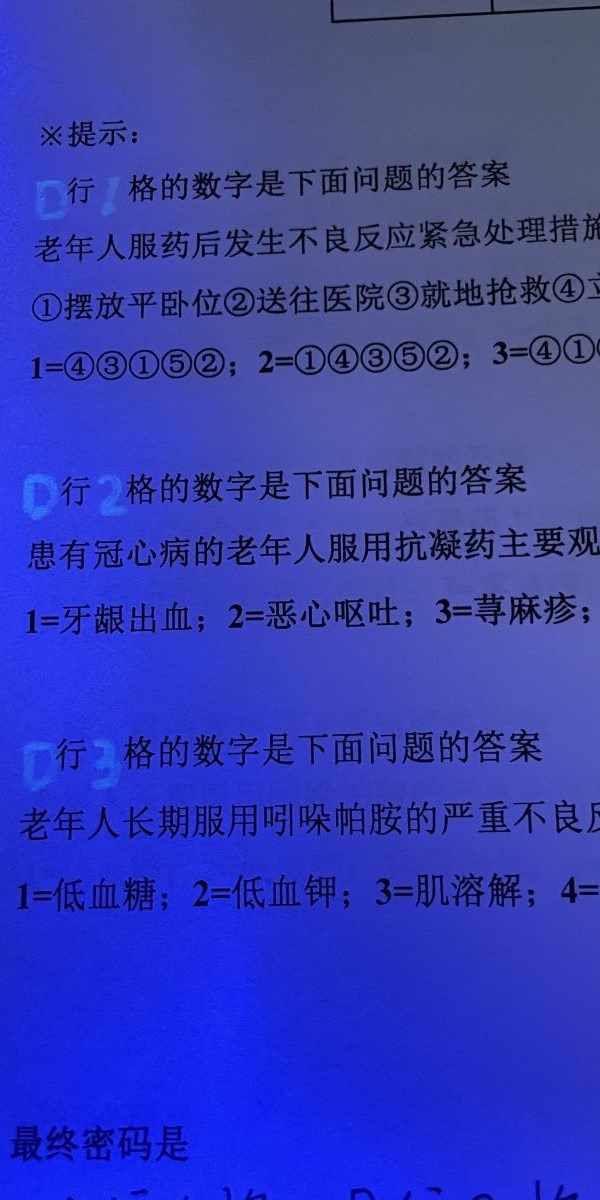


Fig. 3 Only after wearing reading glasses can you see the Sudoku game combined with theoretical knowledge
